# Artificial Intelligence in Clinics: Enhancing Cardiology Practice

**DOI:** 10.31662/jmaj.2024-0190

**Published:** 2024-12-24

**Authors:** Akira Sakamoto, Yutaka Nakamura, Eiichiro Sato, Nobuyuki Kagiyama

**Affiliations:** 1 Department of Cardiovascular Biology and Medicine, Juntendo University Graduate School of Medicine, Tokyo, Japan

**Keywords:** Artificial Intelligence, Cardiovascular Care, Clinic

## Abstract

In recent years, every aspect of the society has rapidly transformed because of the emergence of artificial intelligence (AI) technologies. AI excels not only in image and voice recognition and analysis but also in achieving near-natural conversations through the development of large language models. These technological innovations are steadily being integrated into healthcare settings and can significantly change the way physicians work in clinics in the near future. Patient interviews will predominantly be performed by AI. Physicians will discuss the findings of traditional tests like electrocardiograms and chest X-rays with AI, providing beyond-human interpretation. Additionally, AI is changing areas that have seen little development for a long time, such as auscultation and phonocardiography, and the recognition and quantification of previously challenging observations like the gait analysis. Although barriers to real-world implementation exist, in the near future, a majority of physicians will collaborate with AIs supporting various aspects of clinical practice, consequently enabling more accurate and appropriate diagnosis and treatment of cardiovascular diseases, including ischemic and valvular heart diseases, arrhythmias, and heart failure. This review focuses on AI application in the field of cardiology, specifically on how it can improve the workflow in clinical settings. We examine various examples of AI integration in cardiology to demonstrate how these technologies can lead to more accurate and efficient patient care. Understanding the advancements in AI can lead to more appropriate and streamlined medical practices, which will ultimately benefit both healthcare providers and patients.

## Introduction

For over a century, the medical consultation process in hospitals has followed a traditional flow, i.e., physicians conduct interviews, listen to patients’ complaints, use clinical reasoning, check tests, and then make a diagnosis. Despite some changes, patient assessment has largely been manual. However, a dramatic transformation is now underway with the rise of artificial intelligence (AI), especially applications developed using deep learning techniques ^(1)^. The widespread use of big data and the machine power to handle it have made it possible to perform analyses and predictions that are more powerful than traditional statistical methods ^(2)^. These innovations are increasingly being incorporated into clinical practice and set to expand significantly in the coming years ^(3)^. The impact of AI on medical practice, particularly in the areas of clinical reasoning and image and sound recognition, promises to be profound in both general hospitals and local clinics.

AI supports general practitioners by enhancing their abilities to deduce health conditions from consultations and by assisting in the interpretation of common diagnostic tests, such as electrocardiograms and chest X-rays. However, what does a future with AI-integrated consultations look like? Even before patients step into the examination room, they might undergo an AI preliminary check, which could analyze their complexion and gait, even the sound of their cough, and conduct an initial interview. Based on this information, AI could suggest potential diagnoses and necessary tests for differentiation, which physicians could subsequently use to guide further consultations and test interpretations. This collaborative model between AI and human doctors can potentially become the norm.

This review focuses on the role of AI in cardiology, discussing not only its use in taking medical history, physical examinations, interpreting electrocardiograms, chest X-rays, and echocardiograms, but also its potential in restoring interest in phonocardiograms, a somewhat neglected area, until recently ^(4), (5), (6), (7), (8)^. Cardiology deals with life-threatening conditions, wherein rapid diagnosis and treatment are crucial. Even nonspecialists often face the need to make initial diagnoses of conditions like ST-elevation myocardial infarction or atrial fibrillation. The study explores how AI can be used to improve both the efficiency and the accuracy of cardiological care in general clinics, highlighting examples of literature achievements in the field (Table 1).

**Table 1.** Applications of Artificial Intelligence in Clinic.

Type of test	Year	Authors	Brief summary	AI technology	Model input	Data source (Sample size)	Key findings	Reference
Medical interviews	2020	Harada Y. et al.	An LLM-based, automated medical history-taking system did not reduce waiting time for patients.	LLM	Papers, Journals, Guidelines, Electronic medical records, Public database, etc	Over 50,000 peer-reviewed medical articles, guidelines from the Japanese Society of Internal Medicine and the AMA, major medical journals, epidemiological data from CDC, WHO and others, etc	The system may improve the quality of care by supporting the optimization of staff assignments.	11
Diagnostic dialogue	2024	Tu T. et al.	Diagnostic accuracy of conversational medical LLM optimized for diagnostic dialogue was assessed as higher than that of primary care physicians.	LLM	Multiple-choice medical question answering, expert-curated long-form medical reasoning, electronic health record note summaries, and large-scale transcribed medical conversation interactions	11,450 USMLE multiple-choice style open domain questions with four or five possible answers, 64 long-form medical question answering from MultiMedBench, 65 clinician-written summaries of medical notes from MIMIC-III, 89,027 audio transcripts of medical conversations during in-person clinical visits	This study does not have real-world patients.	14
Assessment of fraility	2024	Mizuguchi Y. et al.	Frailty assessment using ML models created from clinical information and features generated from walking videos by DL is associated with the risk of all-cause death in elderly patients with heart failure.	DL, ML	Walking video and clinical information	417 patients with chronic heart failure over 75-year-old	Excellent agreements between the actual and predicted clinical frailty scale.	17
ECG and heart sound	2023	Shiraga T. et al.	ML models that integrate auscultation and ECG can efficiently detect conditions typically diagnosed via imaging.	ML	Raw PCG data, cropped ECG data, and echocardiography diagnosis	1,052 patients undergoing echocardiography	Patients could be screened for severe AS, severe MR, and LVEF <40%.	22
ECG	2010	Kosmicki DL. et al.	The acoustic cardiographic model can predict LV systolic dysfunction.	ML	ECG and acoustic cardiographic data (S3, S4, and systolic time intervals)	433 patients who had ECG, echocardiography, and BNP	This model outperformed BNP alone for predicting LV systolic dysfunction.	23
PCG	2008	Efstratiadis S. et al.	Assessed the correlation between systolic dysfunction and EMAT.	ML	％EMAT from PCG, findings of echocardiography, and left heart catheter data	25 patients undergoing echocardiography, left-side heart catheterization, and PCG	An abnormal %EMAT was strongly associated with impaired LV dysfunction.	24
ECG	2023	Al-Zaiti S S. et al.	AI outperformed both precision and sensitivity in detecting NSTE-ACS.	ML	Raw ECG data	7,313 patients with chest pain	AI helped correctly reclassify one in three patients.	33
ECG	2019	Attia Z. et al.	AI enabled identification of atrial fibrillation in ECG acquired during normal sinus rhythm.	CNN	Raw ECG data	180,922 patients and 649,931 ECGs	AI identified atrial fibrillation with an AUC of 0.87.	36
ECG	2021	Yao X. et al.	The use of an AI algorithm based on ECGs can enable the early diagnosis of low EF.	CNN	Raw ECG data	22,641 patients without a history of heart failure	More echocardiograms were obtained in the AI-positive ECGs.	37
ECG and echocardiogram	2021	Goto S. et al.	AI models with ECGs enhanced the performance of echocardiography models.	CNN	Raw ECG data and raw echo images	5,495 studies for derivation, 2,247 studies for validation, and 3,191 studies for testing	Echocardiography model performance improved at 67% recall from PPV of 33% to PPV of 74-77%.	38
ECG	2022	Tison GH. et al.	AI-ECG can evaluate HCM status and treatment response.	CNN	Raw ECG data	216 patients diagnosed with HCM	HCM scores by AI-ECG correlated with LV outflow tract gradients and NT-proBNP levels.	39
ECG	2021	Cohen-Shelly M. et al.	AI-ECG can identify patients with moderate or severe AS.	CNN	Raw ECG data	258,607 patients undergoing echocardiography and ECG	The performance of the AI model increased with age and sex (AUC 0.90).	41
X-ray	2024	Bhave S. et al.	AI analysis of X-rays may be useful in the early identification of patients with LV hypertrophy or dilation.	DL	Chest X-ray images	71,589 X-rays from 24,689 patients	The model outperformed all 15 individual radiologists in predicting LV hypertrophy or dilatation.	45
X-ray	2023	Saito Y. et al.	PAWP estimated from X-ray was useful for identifying and monitoring pulmonary congestion.	DL	Chest X-ray images	534 patients admitted for acute heart failure	PAWP calculated by X-ray was significantly associated with higher event rates.	8
X-ray	2021	Homayounieh F. et al.	AI may improve diagnostic performance of radiologists in detecting pulmonary nodules on chest X-ray.	DL	Chest X-ray images	100 X-rays	Junior radiologists saw greater improvement in sensitivity for nodule detection with AI compared with their senior counterparts.	48
X-ray	2024	Weiss J. et al.	AI may help identify individuals at high risk from X-ray when ASCVD risk score cannot be calculated.	DL	Chest X-ray images	8,869 patients with unknown ASCVD risk score and 2,132 patients with known risk score	ASCVD risk of 7.5% or higher as predicted by AI had a higher 10-year risk for MACE after adjustment for risk factors.	52
Echocardiogram	2021	Narang A. et al.	AI allows novices without experience in ultrasonography to obtain diagnosis for evaluation of LV size, LV function, RV size, and pericardial effusion.	DL	Raw echo images	240 patients examined by eight nurses	Nurse and sonographer scans were not significantly different for most parameters.	55
Echocardiogram	2023	He B. et al.	Initial assessments of LVEF by AI was noninferior to assessment by sonographers.	DL	Raw echo images	3,769 exams	The AI saved time for both sonographers and cardiologists. Cardiologists were not able to distinguish between the AI and the sonographer.	57

## System of Medical Interviews

Taking the medical history of a patient is an important technique used by a medical practitioner to get information from the patient and to estimate the diagnosis. As had been said, 70%-80% of patients can be diagnosed through history-taking alone; however, in daily practice, it is sometimes difficult to take a detailed and accurate history due to the limited time for examining each patient and the patient’s inability to convert their own words into medical information.

In recent years, with the development of the information technology, several AI-driven medical history-taking systems that present differential diagnosis have been implemented. In 2019, an automated medical history-taking device, called diagnosis and anamnesis (DIAANA), was developed. It takes a medical history through an interactive questionnaire and presents differential diagnoses. In the emergency department, residents who received this support were able to present differential diagnoses with a greater accuracy than those who did not ^(9)^. However, the development company of this system is now liquidated. Note that systems developed on a commercial basis can become unavailable due to the loss of the development company.

In Japan, a medical interview support system, called Ubie, was developed using a question flowchart-type application based on a Bayesian model. When residents use this system to obtain medical interviews with simulated patients, they are able to make relatively accurate diagnoses and reduce the consultation time overall, albeit some variations between cases ^(10)^. In real patients, although the patient waiting times were not reduced ^(11)^, the incidence of diagnostic errors when the final diagnosis was included in the AI system’s differential diagnosis list was lower, albeit not being statistically significant, than when the final diagnosis was not included in the list ^(11)^. Additionally, diagnostic errors, in which the doctor rejects the correct diagnosis given by AI and in which the doctor accepts the wrong diagnosis given by AI, occurred to the same extent.

Furthermore, advances in the natural language processing technology have also led to the development of various conversational agents, such as chatbots and embodied conversational agents. One of the most famous general-purpose large-scale language model AIs is ChatGPT, which was developed by OpenAI. GPT stands for “generative pretraining transformer,” an advanced language model that uses deep learning techniques to generate human-like responses to natural language input ^(12)^. The GPT collects data from training sources and learns the relationships between various data, thereby statistically estimating the data that should be output next. The training sources include articles published on the internet, websites of medical institutions, and other health information, but are not specifically trained for medical use. Despite that, the latest version (GPT-4) can accurately diagnose 23 of 30 cases on the basis of medical history alone, which is comparable to a doctor’s diagnosis ^(13)^. AMIE was developed as a large-scale language model (LLM)-based AI system optimized for diagnostic dialogue. It was trained on real-world data, such as multiple-choice medical question answers, medical record summaries, and medical conversation records. AMIE can achieve informed and reasoned answers by performing a chain of reasoning-analyzing patient information, generating a response, and refining the response-during patient interaction. In the OSCE format validation, AMIE showed better diagnostic accuracy and behavior compared to primary care physicians ^(14)^.

## Gait Assessment

The human gait can be affected by cardiovascular, neuromuscular, and various other diseases and physical conditions. Previous gait analysis methods diagnosed gait disturbance patterns from a combination of several visually assessed and qualitatively described abnormal findings ^(15)^ or by measuring a limited number of parameters, such as walking distance or time. The results were used to predict disease or diagnose and assess a disease in combination with other laboratory findings. Despite the visual gait analysis being routinely performed by primary care physicians, it requires a great deal of experience and is difficult to standardize. The development of advanced devices and software for quantitative and comprehensive gait analyses in recent years allowed us to obtain more complex and numerous parameters and data compared to the previous years. These technologies have made it possible to evaluate various diseases and physical conditions that could not be evaluated by the conventional qualitative gait analysis.

Two main methods are performed to utilize AI in the gait analysis: one is AI-based motion capture, and the other is AI-based disease prediction and assessment from the large amount of data obtained. Motion capture has been performed using three-dimensional cameras and body markers at the laboratory level, but these are expensive and complex to use in primary care. Currently, several noncontact or simple colored marker-based ^(16)^ systems have been developed that use AI to perform motion capture from video captured by a camera. These systems can estimate the position of joints and key points, gait events (e.g., heel contact and toe-off), spatiotemporal parameters (e.g., stride length and gait speed) and kinematic parameters (e.g., joint flexion and extension angles).

For specific cardiovascular applications, a machine learning-based system was created to assess the clinical frailty scale (CFS) of HF patients by performing gait analysis from videos captured with a smartphone ^(17)^. In this study, videos of walking on an L-shaped walking track, each was 2 m long, were recorded using a smartphone camera. The videos were analyzed using a deep learning-based posture estimation library for estimating the gait parameters. Based on the obtained gait parameters and clinical information, the LightGBM model was used to predict CFS, which showed a good agreement with the actual CFS. In this study, two steps were required: estimation of gait parameters from gait videos and prediction of CFS from gait parameters. Not all processes were automated; hence, developing an application that automates all processes was beneficial. The separation of the two processes allowed for the validation and interpretation of the validity of the CFS prediction using gait parameters. General gait analysis applications are currently often used for disease prediction, but the conditions for capturing gait videos and the gait parameters generated are different for each application. Although the required parameters may differ depending on the disease, there is potential for performing multiple disease predictions from a single gait video by verifying the errors between gait analysis applications and unifying the conditions.

## Auscultation of Heart Sound

Auscultation has long been a cornerstone examination technique for cardiovascular disease screening, enabling a highly noninvasive estimation of structural heart diseases, such as valvular conditions. However, discrepancies in auscultation skills and challenges in quantifying and diagnosing information obtained through auscultation have been noted ^(18), (19)^. Phonocardiography has served as a method for quantifying auscultation, with phonocardiograms becoming practical in the 1950s. However, the advent of echocardiography has significantly diminished the clinical use of phonocardiograms. Today, AI technology advancements have spurred research into evaluating valvular diseases and heart failure through the analysis of phonocardiogram waveforms.

Recent progress in deep learning and signal processing technologies has popularized research on digitized heart sounds. In 2005, the analysis of heart sounds could be used to diagnose mild to moderate aortic stenosis (AS) with a relatively high sensitivity ^(20), (21)^. Moreover, integrating information from 12-lead electrocardiograms with heart sound data has also shown high diagnostic ability for AS, mitral regurgitation, and reduced left ventricular ejection fraction (LVEF) ^(22)^. In applying phonocardiograms for heart failure, Kosmicki et al. demonstrated in 2010 that the AUDICOR system significantly enhanced S3 detection in patients with LVEF below 50% ^(23)^. A model predicting LVEF above 50% using electromechanical activation time (EMAT) was also developed ^(24)^. As a monitoring method in clinical practice, sensors were placed in CRT-D devices ^(25)^, but wearing a LifeVest or a device implantation is required; thus, the development of simpler and less invasive methods is anticipated.

In the respiratory disease field, where auscultation is also a primary diagnostic method, advancements in AI-assisted auscultation are progressing similarly. As reported, the neural network analysis of respiratory sounds can detect wheeze, rhonchi, fine crackles, and coarse crackles with high sensitivity ^(26)^. For fine crackles, quantitative values (fine crackle quantitative values: FCQV) were calculated, demonstrating their association with the diagnosis of idiopathic pulmonary fibrosis and fibrotic findings in HRCT ^(27)^. However, patients with other lung diseases, such as bacterial pneumonia, asthma, and COPD, were excluded, indicating the need for further research for a broader clinical application.

With recent advancements in treatments for cardiovascular diseases, the early diagnosis of valvular diseases and heart failure for facilitating early therapeutic intervention has become increasingly valuable, and the demand for early diagnosis using new devices is expected to grow. A survey of Japanese physicians revealed that 73.4% of them regularly perform auscultation in clinical practice ^(28)^. By a combination with the noninvasive, simple auscultation technique, which can be easily performed not only in examination rooms, but also in home healthcare settings, with the quantification and diagnostic improvements offered by AI technology, further benefits to patients can be realized.

## Electrocardiography

Electrocardiography (ECG) is a highly convenient, minimally invasive, and cost-effective test that can be performed with high reproducibility, making it widely accessible in most clinics. However, interpreting ECGs can be challenging for nonspecialists. Even specialists often rely on comparisons with past records and their experience to judge subtle changes, which can sometimes lead to interpretation uncertainty ^(29)^.

ECG already provides automated diagnostic capabilities, but these are limited to identifying findings on the ECG itself rather than diagnosing the underlying disease. AI can process and analyze more data than a human can, offering results that surpass traditional automated diagnostics, even making predictions about the disease itself.

Acute coronary syndrome (ACS) is one of the most significant cardiovascular diseases encompassing a wide range of conditions from unstable angina to acute myocardial infarction ^(30)^. Early therapeutic intervention is crucial, and ECG is an indispensable test in the diagnostic process. Efforts for predicting ACS from ECG changes outside of hospitals have been explored. Zaiti et al. developed a model using random forest algorithms to predict ischemic heart disease in patients without ST-elevation at initial triage by selecting 73 morphological features from the ECG. In an external validation, this model achieved an area under the curve (AUC) of 0.87 (95% confidence interval (CI), 0.85-0.90), outperforming existing systems and clinicians. This suggests that using AI models can improve initial triage in emergencies, stratify patients who need immediate treatment, and potentially enhance clinical outcomes by directly affecting the ACS prognosis ^(31), (32), (33)^.

Atrial fibrillation (AF) can lead to severe neurological sequelae or be fatal because of thromboembolism; however, early detection has been challenging, especially in asymptomatic or paroxysmal cases. Several models have been reported to predict AF from ECG. Notably, Attia et al. developed a convolutional neural network (CNN) model predicting asymptomatic paroxysmal AF from ECGs recorded during a normal sinus rhythm. Their study, which was published in The Lancet, demonstrated a high predictive performance with an AUC of 0.87 (95% CI, 0.86-0.88). This model was trained on a large dataset of 649,931 ECGs from 180,922 patients and effectively identified patients with AF history using only a single 10-s, 12-lead ECG recorded during sinus rhythm. The study highlights the potential of AI in providing a rapid, inexpensive, and noninvasive method for AF screening at the point-of-care, which could significantly affect the management of patients at AF risk ^(34), (35), (36)^.

Models for predicting cardiac function and diseases from ECG findings have also been reported, identifying conditions, such as reduced left ventricular systolic function, hypertrophic cardiomyopathy, cardiac amyloidosis, and AS. Yao et al. developed an algorithm based on ECG to identify patients with LVEF ≤50%, demonstrating that the early diagnosis of reduced left ventricular ejection fraction is possible. In a study involving 22,641 individuals without a history of heart failure, the group analyzed that using the algorithm showed an increased diagnosis of LVEF ≤50% within 90 days compared to the control group ^(37), (38), (39), (40), (41)^. The implementation of diagnostic applications with such capabilities would enable clinic physicians, particularly those not specialized in cardiology, to effectively use ECGs to guide appropriate treatment and ensure that critical diseases are not overlooked.

## Chest X-ray

Like electrocardiography, chest X-ray (CXR) is a simple and classic test available in many clinics. It is highly useful because it can detect changes related to heart failure, such as cardiac enlargement, pleural effusion, and pulmonary congestion, and assist in differentiating conditions like lung cancer and pneumonia. Additionally, a comparison with past images allows for the detection of temporal changes, making it an indispensable test in heart failure diagnosis. AI-based image recognition in CXR captures subtle changes that were previously difficult for humans to perceive, potentially leading to a more accurate disease diagnosis.

Several studies have reported on predicting clinical findings from CXR images ^(42), (43), (44), (45)^. Saito et al. evaluated the prognostic value of the estimated pulmonary arterial wedge pressure (ePAWP) derived from CXRs using a deep learning model in patients with acute decompensated heart failure (ADHF). Higher ePAWP at discharge was significantly associated with increased risks of all-cause mortality and heart failure rehospitalization. The findings suggest that using chest X-rays to estimate ePAWP can be a valuable tool for assessing pulmonary congestion and predicting clinical outcomes in ADHF patients ^(7), (46)^.

Similarly, AI has shown promise in enhancing the diagnostic accuracy of radiologists. In a multicenter study, an AI algorithm improved radiologists’ accuracy in detecting pulmonary nodules on chest X-rays. The study involved 100 PA chest radiographs reviewed by radiologists with and without AI assistance. The AI-aided interpretation significantly increased the detection sensitivity and specificity, especially benefiting junior radiologists by reducing missed nodules and false positives ^(47), (48)^.

Recent studies reported multiple attempts to directly predict prognosis from CXR images ^(49), (50), (51)^. Weiss et al. developed a CNN model to predict the 10-year risk of future cardiovascular events from routine CXR images, achieving a performance comparable to that of the existing risk score (ASCVD risk score) for predicting the statin therapy efficacy. This model was also able to predict statin efficacy, even when the existing risk score cannot be evaluated ^(52)^. This study highlights a new potential for predicting cardiovascular risk using chest X-ray images and emphasizes the improvement in the accuracy and usefulness of prognostic predictions in clinical practice through the AI technology.

## Echocardiography

Echocardiography is essential in cardiology. It provides critical insights into the wall motion, valvular disease, and hemodynamics of heart failure ^(53)^. Despite its utility, it is often seen as specialized and less reproducible, posing challenges for general clinics. However, its noninvasive nature and rich information make it valuable.

Recent AI advancements have significantly affected this field. AI technologies assist with image acquisition ^(54), (55)^ and automated measurement and analysis ^(56), (57)^. AI is also integrated with other modalities for enhanced disease diagnosis and pathophysiological analysis ^(38), (58), (59)^.

EchoNet-Dynamic by Ouyang et al. predicted LVEF with a mean absolute error of 4.1% and accurately classified heart failure with reduced ejection fraction with an AUC of 0.97. Studies showed that AI significantly reduces the percentage of LVEF readings adjusted by specialists from 27.2% (technician analysis) to 16.8% (AI analysis, p < 0.001). AI also reduced the time required for the analysis and specialist review, suggesting that it can alleviate barriers to echocardiography in clinics ^(57)^.

Echocardiography’s utility extends beyond cardiology, aiding in diagnosing gastrointestinal diseases. As reported, it is effective in diagnosing nonalcoholic fatty liver disease (NAFLD) and pediatric appendicitis, as well ^(60), (61), (62)^.

AI advancements are set to reduce resistance to echocardiography and address time constraints, leading to broader adoption in clinics. This integration promises enhanced diagnostic accuracy and streamlined workflows, improving patient outcomes.

## Development Potential for the Future

Efforts of utilizing AI in various diagnostic tests are advancing, and it is anticipated that in the future, services comparable to a physician’s examination can be provided at home. The development of various wearable devices has been remarkable in recent years ^(63)^. Accordingly, AI systems might be developed to use the biometric data obtained from these devices for health management, early disease detection, and prompting medical consultations. These systems can be beneficial not only for healthy individuals, but also for managing chronic diseases, such as supporting home care for chronic heart failure patients ^(64)^. By monitoring the weight gain and the blood pressure fluctuations and interacting with AI to detect the early signs of nocturnal dyspnea or worsening leg edema, early medical intervention can prevent heart failure hospitalizations, thereby improving long-term outcomes ^(65)^. The benefits of such monitoring systems will be significant, especially in remote areas where clinics are limited, and specialists are scarce ^(4)^. Not only for home use, but also in primary care settings, AI is expected to have the power to transform medical practice. Sengupta et al. developed a deep learning model for predicting high-sensitivity cardiac troponin I (hs-cTnI) levels in ACS patients using a wrist-worn transdermal infrared spectroscopic sensor (transdermal-ISS). Evaluating the accuracy of the machine learning algorithm using optical data on 238 hospitalized ACS patients, the model demonstrated a high diagnostic accuracy in both internal and external validation cohorts, with AUCs of 0.90 and 0.92, respectively. This finding suggests that rapid diagnosis without blood tests is possible, highlighting the potential of this approach as a point-of-care tool in ACS management ^(66)^.

## Future Perspective and Potential Barrios

**Figure 1.**
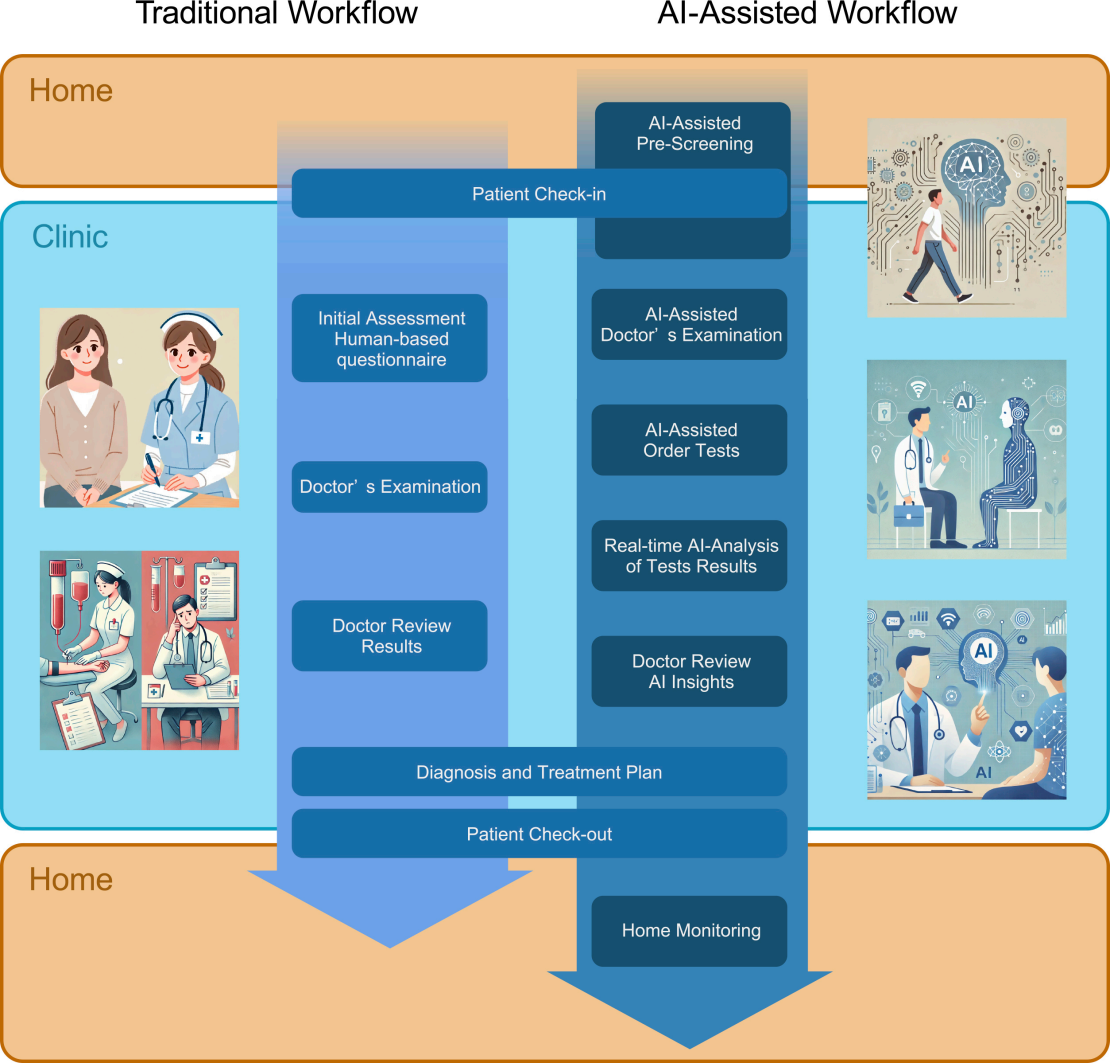
Workflow of traditional and AI-assisted clinic Comparison of the traditional workflow and AI-assisted workflow in a clinical setting. The traditional workflow (left) follows a linear process from patient check-in to check-out, involving several manual steps, such as initial assessment, doctor’s examination, and review of test results. The AI-assisted workflow (right) incorporates AI at multiple stages, including AI-assisted prescreening, doctor’s examination, test ordering, and real-time analysis of test results, providing AI insights for the doctor. Additionally, the AI-assisted workflow includes home monitoring, extending care beyond the clinic.

In the near future, patients will be interviewed by AI from the moment they walk into a hospital, even from when they are at home. Before entering the consultation room, the gait assessment will elicit a range of information and screen for CFS and other conditions. Heart sounds, ECG, and CXR will immediately point out the possibility of cardiac and respiratory diseases requiring a close examination or a hidden AF. AI-assisted echocardiography will allow noncardiac clinics to perform and analyze echocardiograms. By integrating these results, physicians will be able to make more rational and accurate decisions and improve patient outcomes. Home monitoring will help patients manage their disease status and suggest the timing of their next visit appropriately without bothering the patient (Figure 1).

The technologies for realizing AI-assisted medicine are gradually coming together, as mentioned above. However, there are still several challenges to overcome before realizing it. There might be no basis for the diagnosis made by the AI, and in the language model, there is a possibility of creating hallucinations or false information. Therefore, the medical rationale must be re-examined when making a final diagnosis based on the differential diagnoses provided by the AI. Additionally, as AI has learned based on past information, it may reproduce ethically incorrect responses based on race, gender, etc. ^(67)^. Fortunately, the advent of explainable AI (XAI) is making it increasingly possible to understand the reasoning behind AI outputs, offering hope for future improvements. Furthermore, in the case of language models, information related to patient privacy could be learned, which requires appropriate anonymization and/or learning suppression ^(68)^. Other concerns include the digital divide, where individuals with lower digital literacy or limited access to technology may be disadvantaged, and the potential for adversarial attacks that could disrupt machine learning models. Addressing each of these issues will be crucial to enhancing trust in AI.

The emergence of large language models like ChatGPT has revolutionized the AI landscape. These models are well-suited for processing images and audio, as their accuracy improves dramatically with more data accumulation. Therefore, even if current AI capabilities are insufficient, continuous data collection could lead to highly practical AI for image processing in the near future.

As technological innovations progress, the role of physicians will involve collaborating with and utilizing AI while bearing ultimate responsibility and addressing ethical issues. While AI might free physicians from memory-based tasks and routine activities, a deep understanding of medicine, insight, and effective patient communication will remain as an essential skill for a physician as supervisors of such AI and ultimate managers of patient decision-making.

## Conclusion

With the advancement of AI, the environment surrounding traditional medical care is undergoing a significant transformation. This revolution is expected to soon extend to cardiology practices in clinics, potentially leading to higher-quality healthcare and heralding a new era. To integrate these new technologies effectively, it is crucial that both healthcare providers and patients understand and adapt to these AI-driven innovations, demonstrating the necessary flexibility to embrace these changes.

## Article Information

### Conflicts of Interest

Dr. Kagiyama receives research grants from EchoNous, Inc. and AMI, Inc. and speaker honoraria from Eli Lilly, Novartis, Otsuka Pharmaceutical, and Boehringer-Ingelheim outside this work and is affiliated with a department funded by Paramount Bed, Ltd. The other authors declare that there are no conflicts of interest.

### Sources of Funding

This work was supported by JSPS Kakenhi grant number 21K18086.

### Author Contributions

Conceptualization: AS and NK

Supervision: NK

Writing: AS, YN, ES, and NK
